# Cross sectional study to assess the accuracy of electronic health record data to identify patients in need of lung cancer screening

**DOI:** 10.1186/s13104-018-3124-0

**Published:** 2018-01-10

**Authors:** Allison M. Cole, Bethann Pflugeisen, Malaika R. Schwartz, Sophie Cain Miller

**Affiliations:** 1 Department of Family Medicine, University of Washington, Box 354696, Seattle, WA 98195-4696 USA; 2 MultiCare Institute for Research and Innovation, 314 Martin Luther King Jr. Way Suite 402, Tacoma, WA 98405 USA; 3 University of Washington School of Medicine, Box 354696, Seattle, WA 98195-4696 USA

**Keywords:** Electronic health records, Lung cancer, Cancer screening, Primary care

## Abstract

**Objective:**

Lung cancer is the leading cause of cancer death in the United States [Siegel et al. in CA Cancer J Clin 66:7–30, 1]. However, evidence from clinical trials indicates that annual low-dose computed tomography screening reduces lung cancer mortality [Humphrey et al. in Ann Intern Med 159:411–420, 2]. The objective of this study is to report results of a study designed to assess the sensitivity, specificity, and positive and negative predictive value of an electronic health record (EHR) query in comparison to patient self-report, to identify patients who may benefit from lung cancer screening. Cross sectional study comparing patient self report to EHR derived assessment of tobacco status and need for lung cancer screening. We invited 200 current or former smokers, ages 55–80 to complete a brief paper survey. 26 responded and 24 were included in the analysis.

**Results:**

For 30% of respondents, there was not adequate EHR data to make a lung cancer screening determination. Compared to patient self-report, EHR derived data has a 67% sensitivity and 82% specificity for identifying patients that meet criteria for lung cancer screening. While the degree of accuracy may be insufficient to make a final lung cancer screening determination, EHR data may be useful in prompting clinicians to initiate conversations with patients in regards to lung cancer screening.

## Introduction

Lung cancer is the leading cause of cancer death in the United States [1]. However, evidence from clinical trials indicates that annual low-dose computed tomography (LDCT) screening reduces lung cancer mortality [2]. The U.S. Preventive Services Task Force (USPSTF) recommends annual LDCT screening for patients ages 55–80 who have a 30-pack year smoking history and either currently smoke or have quit smoking in the past 15 years [3].

The widespread adoption of electronic health records (EHRs) by primary care providers and health systems, and meaningful use incentives, which encourage using the EHR to document patients’ tobacco status, create opportunities to implement EHR-based clinical decision support tools to promote appropriate lung cancer screening [4–7]. Systematic EHR data queries can also be used to identify populations of patients that may benefit from lung cancer screening.

While the majority of primary care providers routinely document tobacco use status in the EHR, the frequency and accuracy of additional tobacco status details, such as amount smoked and years smoked, are ambiguous [8, 9]. The objective of this study is to assess the sensitivity, specificity, and positive and negative predictive value of an EHR query in comparison to patient self-report, to identify patients who may benefit from lung cancer screening based on the published USPSTF screening recommendations.

## Main text

### Methods

#### Setting

This study was conducted in a large, community-based multispecialty healthcare system in the Pacific Northwest. Approximately 55% of patients are insured through Medicare or Medicaid, 40% commercial insurance, and 5% are self-pay. All study procedures were reviewed and approved by the Human Subjects Institutional Review Board.

#### Data sources

From the shared EHR system, we identified patients ages 55–80 who had been seen at primary care clinics between 5/1/13 and 4/30/15 and for whom the physician had recorded smoking status as current or former. We limited the initial inclusion criteria to current or former smokers because the goal of the study was to assess the accuracy of smoking status documentation for the indication of lung cancer screening, and non-smokers would not be eligible for lung cancer screening. We randomly sampled 200 individuals for invitation to participate. We extracted data from Epic (Verona, Wisconsin) Enterprise Data Warehouse. Manipulation and sampling was performed in the R statistical computing environment (Vienna, Austria). To all 200 patients, we mailed a study information sheet, informed consent form and a six-question, single-page paper questionnaire. Patients were invited to complete the questionnaire and informed consent paperwork and return them with the enclosed self addressed, stamped envelope. Patients were offered a $5 Starbucks gift card for completing the survey and consent document. The EHR smoking status, packs per day, years smoked and start/quit dates (when available) were linked at the patient level for patients with completed surveys and consent paperwork. All data were de-identified and securely transferred to the University of Washington for analysis and interpretation.

#### Variables

##### Smoking status

From information on the paper survey, a current smoker was defined as anyone who answered “yes” to the question regarding smoking now. Anyone who answered “no” to the question about smoking now, but “yes” to smoking in the past was defined as a former smoker. From the EHR, structured data fields on tobacco status (categorized as current/former/never) were used.

##### Years smoked

Years smoked was calculated by subtracting the year they began smoking from the year they quit smoking. If they were a current smoker, the year they began smoking was subtracted from 2015, the year the data was collected. From the EHR we obtained available data on the number of years the patient had smoked.

##### Pack-Years

A pack-year is defined as 20 cigarettes (a typical pack) smoked every day for 1 year. From the paper survey, pack-years were calculated by multiplying the packs per day by the number of years smoked. From the EHR, we obtained data from the “packs per day” data field and multiplied this number by the number in the “years smoked” field to calculate pack-years.

##### Lung cancer screening

For both paper survey data and EHR data, patients with a 30 + pack-year smoking history who were current smokers or who quit smoking between 2000 and 2015 were identified as eligible for lung cancer screening.

#### Statistical analysis

We calculated frequencies of responses to all questions. We calculated the sensitivity, specificity, positive predictive value (PPV), and negative predictive value (NPV) for EHR data, using patient self-reports as the comparison. We conducted all analyses using Stata 14.1.

## Results

Of the 200 patients invited to participate, 26 responded (13%). Two patients returned incomplete surveys, which left 24 participants remaining in this analysis.

Half of the participants were current smokers and half were former smokers (Table 1). Compared to participant self-report, fewer patients were categorized by the EHR data as having smoked ≥ 30 years (63% vs. 29%). Based on self-report, 46% of patients were eligible for lung cancer screening, compared to only 25% of participants based on EHR data. Overall, 83% of respondents believed they should receive lung cancer screening.

**Table 1:** Characteristics of patients (N = 24)

Characteristic	Patient self-report	EHR derived
	*N (%)*	*N (%)*
Age		
50–59	8 (33.33)	Not assessed
60–69	8 (33.33)	
70–79	8 (33.33)	
Smoking status		
Current cigarette smoker	12 (50.00)	12 (50.00)
Former cigarette smoker	12 (50.00)	12 (50.00)
Years quit		
< 15	10 (41.67)	11 (45.83)
≥ 15	2 (8.33)	1 (4.17)
Missing	12 (50.00)	12 (50.00)
Packs per day		
< 1 pack	12 (50.00)	14 (58.33)
1–2 packs	12 (50.00)	10 (41.67)
Years smoked		
≤ 10	1 (4.17)	3 (12.50)
11–20	0 (0)	2 (8.33)
21–30	2 (8.33)	1 (4.17)
> 30	21 (87.50)	11 (45.83)
Missing	0 (0.00)	7 (29.17)
Pack-years		
< 30	9 (37.50)	10 (41.66)
≥ 30	15 (62.50)	7 (29.17)
Missing	0 (0.00)	7 (29.17)
Lung cancer screening indicated		
Yes	11 (45.83)	6 (25.00)
No	13 (54.17)	11 (45.83)
Unable to determine	0 (0.00)	7 (29.17)
Patient believes lung cancer screening needed		
Yes	20 (83.33)	Not assessed
No	4 (16.67)	

Only the 17 patients with adequate EHR data to assess lung cancer screening need were included in the analysis to assess agreement between EHR and self-report.). The PPV of the EHR data was 66.67%, and the NPV was 81.82% (Table 2).

**Table 2:** Accuracy of EHR data-based determination of need for lung cancer screening, compared to patient self-report (N = 17)

Sensitivity	66.67% (95% CI 22.28–95.67)
Specificity	81.82% (95% CI 48.22–97.72)
Positive predictive value	66.67% (95% CI 22.28–95.67)
Negative predictive value	81.82% (95% CI 48.22–97.72)

## Discussion

In this pilot study, we found that EHR data had a 66.7% PPV and 81.8% NPV for identifying patients eligible for lung cancer screening. In this sample, there was inadequate information in the EHR to determine the need for lung cancer screening for almost one-third of the participants. Missing data remains a significant problem when conducting research with EHR systems [10]. Strategies to improve documentation of tobacco use in EHRs include evidence-based prompts to guide medical assistants to identify smokers and remind clinicians to deliver tobacco cessation recommendations [11, 12]. Similar to our reported sensitivity and specificity, the reported sensitivity of EHR data for identifying patients who have completed cancer screening tests ranges from 55% for colorectal cancer to 96% for cervical cancer [13]. The results of this pilot study are useful for future work to estimate necessary sample size and recruitment populations for efforts to implement and evaluate lung cancer screening in healthcare systems. While the degree of accuracy in EHR data may be insufficient to make a final lung cancer screening determination, EHR data may be useful in prompting clinicians to initiate conversations with patients in regards to lung cancer screening.

## Limitations

This study was conducted single health system and included a small number of participants, which limits the generalizability of our findings. Our initial sample included only adults who had EHR evidence of current or former smoking. Thus, we are not able to estimate the prevalence in a health system population of patients who may meet eligibility for lung cancer screening. Future work to include all patients age 55–80 which would allow one to assess the accuracy of smoking status overall as well as the proportion of a health system population in need of lung cancer screening, both important considerations when planning large scale lung cancer screening programs. Our study also used patient self-report as the standard for assessing tobacco status and individuals tend to underreport tobacco use. More accurate methods of assessing tobacco use, such as measurement of exhaled carbon monoxide, may be difficult to implement in routine healthcare settings and only self report can establish non-recent tobacco history, such as for former smokers [14]. Efforts to improve the accuracy of years smoked and the accuracy and frequency of documentation of packs per day smoked would improve the overall accuracy of EHR-derived lung cancer screening recommendations.

## Abbreviations

LDCT: low dose computed tomography
EHR: electronic health record
USPSTF: United States Preventive Services Task Force
PPV: positive predictive value
NPV: negative predictive value
